# Pheochromocytoma-Induced Hypertension After Traumatic Brain Injury

**DOI:** 10.7759/cureus.44161

**Published:** 2023-08-26

**Authors:** Justin L Weppner, Justin Tu, Ayub Khan, Justin S Raucheisen

**Affiliations:** 1 Physical Medicine and Rehabilitation, Carilion Clinic, Roanoke, USA; 2 Physical Medicine and Rehabilitation, Emory University, Atlanta, USA; 3 Medical School, West Virginia School of Osteopathic Medicine, Lewisburg, USA

**Keywords:** motor vehicle accidents, brain injury rehab, traumatic brain injury, hypertension, pheochromocytoma

## Abstract

A 23-year-old female presented to the emergency department (ED) after sustaining a motor vehicle accident and subsequent loss of consciousness. In the ED, the patient was hemodynamically stable and was appropriately discharged with a diagnosis of mild traumatic brain injury. The patient presented 10 days post-injury to the outpatient brain injury clinic with complaints of headache, anxiety, and dizziness, with an elevated blood pressure of 160/100 mmHg. Initial head imaging, drug screen, complete blood count, and complete metabolic panel were unremarkable, however, urine and plasma metanephrines were found to be elevated. Abdominal computed tomography imaging revealed a pheochromocytoma, and the patient was adequately treated with medication and adrenalectomy with complete resolution of symptoms. Existing literature has indicated that stress and physical trauma can contribute to the escalation of pheochromocytoma symptoms in previously asymptomatic individuals; here, the stress and trauma stemming from an automobile accident and mild traumatic brain injury may have precipitated the onset of pheochromocytoma symptoms in the patient. Symptoms of pheochromocytoma can align with those commonly observed after traumatic brain injury (TBI), encompassing headaches, anxiety, and dizziness. Our case demonstrates the need for clinicians to consider the presence of pheochromocytoma in a post-traumatic brain injury patient.

## Introduction

Pheochromocytoma is a rare neuroendocrine tumor arising from chromaffin cells within the adrenal medulla. The development of pheochromocytoma can be idiopathic or linked to mutations within genes such as *RET*, *VHL*, and *NF1* [1]. The occurrence of pheochromocytoma is approximately 0.6 cases per 100,000 people per year [2]. Symptoms arise from the tumor's secretion of catecholamines including epinephrine and norepinephrine, and include headaches, palpitation, anxiety, profuse sweating, and hypertension. Pheochromocytoma presents with sympathetic symptoms that mimic several pathologies, and a misdiagnosis of pheochromocytoma can have lethal consequences due to the effects of excessive circulating catecholamines or the development of metastatic disease [3]. Therefore, clinicians must consider pheochromocytoma in a wide variety of clinical contexts, including trauma from a motor vehicle accident. In this case, we describe the clinical presentation, diagnostic evaluation, and treatment course of pheochromocytoma in a post-traumatic brain injury (TBI) patient with hypertension.

## Case presentation

A 23-year-old woman with an unremarkable medical, family, and psychosocial background arrived at the emergency room following a motor vehicle collision that resulted in hitting her head. The patient experienced a brief period of unconsciousness lasting around 10 minutes, as well as a Glasgow Coma Scale (GCS) score of 14, as assessed by emergency medical services. This score improved to 15 within an hour of the incident and the patient met the American Congress of Rehabilitation Medicine Diagnostic Criteria for Mild Traumatic Brain Injury [4]. In the emergency room, the patient’s evaluation included unremarkable vital signs, a physical exam, and non-contrast head computed tomography (CT). Ten days post-injury, however, the patient outpatient brain injury clinic and was noted to have headaches, anxiety, and dizziness. Vital signs at that time revealed a blood pressure of 160/100 mmHg (at baseline her blood pressure had been normotensive), and her physical examination remained unremarkable with no focal neurological deficits.

**Figure 1:**
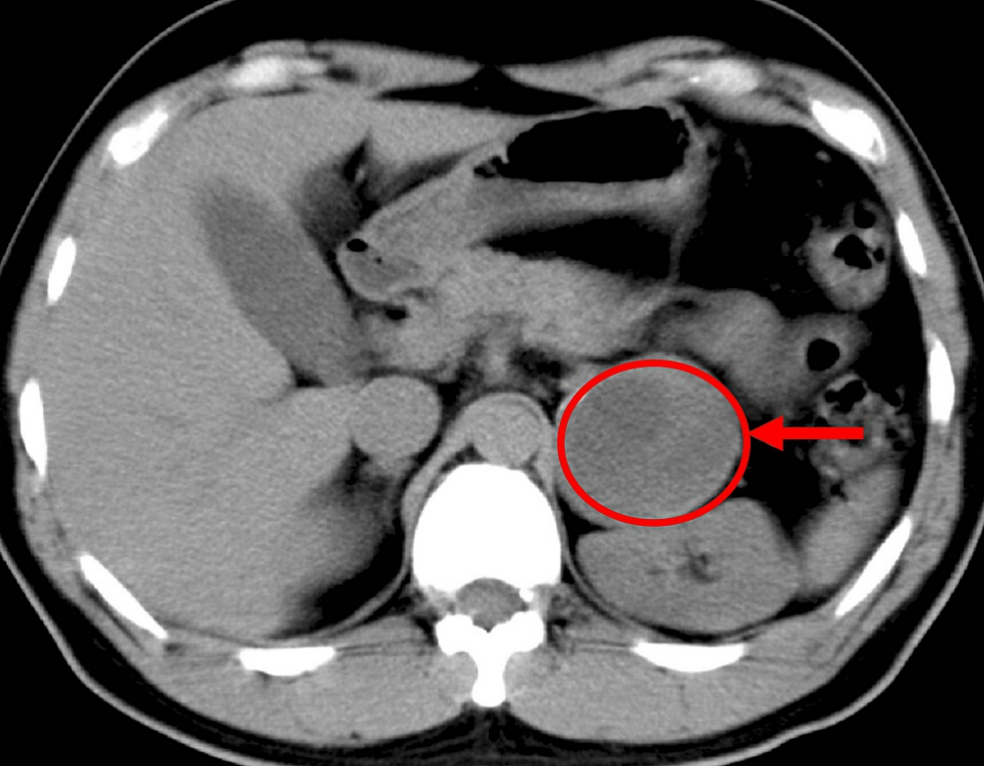
CT of the abdomen An axial view of the CT of the abdomen shows pheochromocytoma located in the left adrenal gland (red circle and arrow).

Initial laboratory evaluation including complete blood count, comprehensive metabolic panel, thyroid stimulating hormone, free thyroxine, luteinizing hormone, follicle-stimulating hormone, insulin-like growth factor 1 (IGF-1), and cortisol, all of which were unrevealing as to an etiology for the patient’s symptoms. A urine drug screen did not show any substances that would be contributing to the patient’s presentation. However, plasma and urine-fractionated metanephrines were both elevated, with urine-fractionated metanephrines at 1700 mcg/24 hours (reference <400 mcg/24 hours) and plasma-fractionated metanephrines at 2.2 nmol/L (reference < 0.50 nmol/L). Imaging with an abdominal CT demonstrated a 6.1 x 5 x 5.8 cm left adrenal mass consistent with pheochromocytoma (Figure 1).

Antihypertensives were started for this patient. Following pharmacologic management, surgery was consulted, and the patient underwent a left adrenalectomy that resulted in blood pressure normalization and resolution of the patient’s symptoms.

## Discussion

Classically, high systemic blood pressure after TBI centers around a catecholamine excess state, with injury to the brain parenchyma triggering pathways of catecholamine release through regional injury to the brain, elevation in intracranial pressure (ICP), and activation of the lower brain and hypothalamic neuroendocrine pathways. While the initial catecholamine response and systemic hypertension could be initially protective by maintaining cerebral perfusion pressure (CPP), secondary brain damage can be aggravated by catecholamine-induced hypertension with aggravation of vasogenic edema and intracranial hypertension [5]. Existing literature elucidates that stress and physical trauma can play a role in intensifying pheochromocytoma symptoms and are associated with an excess release of catecholamines in individuals who were previously without symptoms [6-8]. This is consistent with our case in which the patient did not experience any symptoms or vital instability prior to the automobile accident. The stress and physical trauma arising from the accident and mild TBI may have aggravated the existing pheochromocytoma and triggered the manifestation of the patient's symptoms. 

In addition to hypertension, some post-TBI patients can demonstrate associated paroxysmal tachycardia, tachypnea, and hyperthermia in response to external stimuli, recently termed paroxysmal sympathetic hyperactivity (PSH) [9]. Specific regions associated with increased blood pressure after TBI include the orbitofrontal region and the posterior hypothalamus. If the orbitofrontal cortex is injured after TBI, there is dysregulated inhibition of the sympathetic system, whereas if the posterior hypothalamic region is stimulated, the consequence is increased blood pressure and heart rate with inhibition of baroreceptor bradycardia and promotion of sympathetic constrictor activity [10]. Our patient's presentation, imaging, and elevated metanephrines are more consistent with pheochromocytoma symptoms.

While it is important to consider anterior pituitary or posterior hypothalamic dysfunction, medications, or renovascular disease as causes of headache, dizziness, and elevated blood pressure post-TBI, it is essential to rule out a pheochromocytoma in any patient with signs and symptoms of excessive catecholamine production. Trauma alone can provoke an unsuspected pheochromocytoma, as well as induction of anesthesia or surgical procedures [10]. With the release of catecholamines into the circulation with the provocation of a pheochromocytoma, the alpha-adrenergic effects of catecholamines produce intense vasospasm and hypertension, whereas the beta-adrenergic effects produce vasodilation, diaphoresis, and tachycardia [11]. Historically, with regards to diagnosis, 24-hour urinary excretion of catecholamines and total metanephrines was used, but plasma fractionated metanephrines have been proposed as an equally effective, if not superior, test for biochemical diagnosis of pheochromocytoma [12]. Upon diagnosis, treatment of hypertensive crisis requires the administration of the long-lasting alpha-blocker phenoxybenzamine or more selective alpha-blockers such as prazosin or doxazosin. Additionally, beta-blockers can be administered when tachyarrhythmia occurs, but should never be administered alone as unopposed vasoconstriction would occur with a resultant hypertensive crisis [11]. In the event of non-malignant pheochromocytoma, surgical removal of the tumor is the primary treatment of pheochromocytoma via laparoscopic adrenalectomy or open surgery for larger tumors.

## Conclusions

Ultimately, there is a broad differential diagnosis that exists regarding hypertension after a motor vehicle collision with TBI. Typically, hypertension occurring post-TBI is in the setting of catecholamine excess due to regional injury in the brain. Thus, it is sensible to perform the necessary imaging to rule out any new intra/extracranial pathology. Further, certain lab work could identify disruptions of various endocrinologic axes due to the traumatic brain injury, or medication side effects. Nonetheless, in the event that the conducted laboratory tests and imaging fail to reveal an underlying cause, it becomes crucial to contemplate less common origins of hypertension following traumatic injury, with a focused emphasis on potential catecholamine excess. Pheochromocytomas can be provoked by trauma and lead to symptoms overlapping with what would be seen after TBI, including headaches, anxiety, and dizziness. Therefore, in patients who present with new onset hypertension following a TBI, it is imperative that clinicians consider pheochromocytoma as a differential.
